# The first direct evidence of a Late Devonian coelacanth fish feeding on conodont animals

**DOI:** 10.1007/s00114-017-1455-7

**Published:** 2017-03-10

**Authors:** Michał Zatoń, Krzysztof Broda, Martin Qvarnström, Grzegorz Niedźwiedzki, Per Erik Ahlberg

**Affiliations:** 1 Faculty of Earth Sciences, University of Silesia, Będzińska 60, 41-200 Sosnowiec, Poland; 2 Department of Organismal Biology, Uppsala University, Norbyvägen 18A, 752 36 Uppsala, Sweden

**Keywords:** Coelacanth, Conodonts, Gut content, Coprolite, Devonian

## Abstract

We describe the first known occurrence of a Devonian coelacanth specimen from the lower Famennian of the Holy Cross Mountains, Poland, with a conodont element preserved in its digestive tract. A small spiral and phosphatic coprolite (fossil excrement) containing numerous conodont elements and other unrecognized remains was also found in the same deposits. The coprolite is tentatively attributed to the coelacanth. Although it is unclear whether the Late Devonian coelacanth from Poland was an active predator or a scavenger, these finds provide the first direct evidence of feeding on conodont animals by early coelacanth fish, and one of the few evidences of feeding on these animals known to date. It also expands our knowledge about the diet and trophic relations between the Paleozoic marine animals in general.

**Electronic supplementary material:**

The online version of this article (doi:10.1007/s00114-017-1455-7) contains supplementary material, which is available to authorized users.

## Introduction

Coelacanths are sarcopterygian fishes which have a long evolutionary history ranging back to the Early Devonian (Zhu et al. 2012). The group comprised numerous species in the geological past, but today is represented by the single genus *Latimeria*, first discovered in 1938 in the Indian Ocean. Although the extant *Latimeria* occupies a deep-sea habitat, fossil coelacanths inhabited a range of different palaeoenvironments and evidently occupied various ecological niches, as indicated by their occurrence in different facies and their morphological disparity (Friedman and Coates 2006; Wendruff and Wilson 2012). Despite growing knowledge about fossil coelacanth diversity, disparity, anatomy, and even ontogeny (Wendruff and Wilson 2012; Gess and Coates 2015), there is still insufficient data on some important palaeobiological aspects, including diets. Gut contents are an important source of direct evidence on food gathering by fossil organisms, including fishes. These, however, are extremely rare in fossil coelacanths. To date, recognizable gut contents have been recorded in only two species. Lund and Lund (1984, 1985) found scales and even an intact paleostomatopod shrimp in the species *Caridosuctor populosum* from the Lower Carboniferous Bear Gulch Limestone of MT, USA. Clement (2005) found a ganoid scale, arthropod appendages, and a crushed, incomplete crustacean within the body cavity of *Wenzia latimerae* from the Upper Jurassic of France. Recently, Yabumoto and Brito (2013) reported some unrecognizable stomach contents in the mawsoniid coelacanth *Axelrodichthys araripensis* from the Lower Cretaceous Crato Formation of Brazil.

**Fig. 1:**
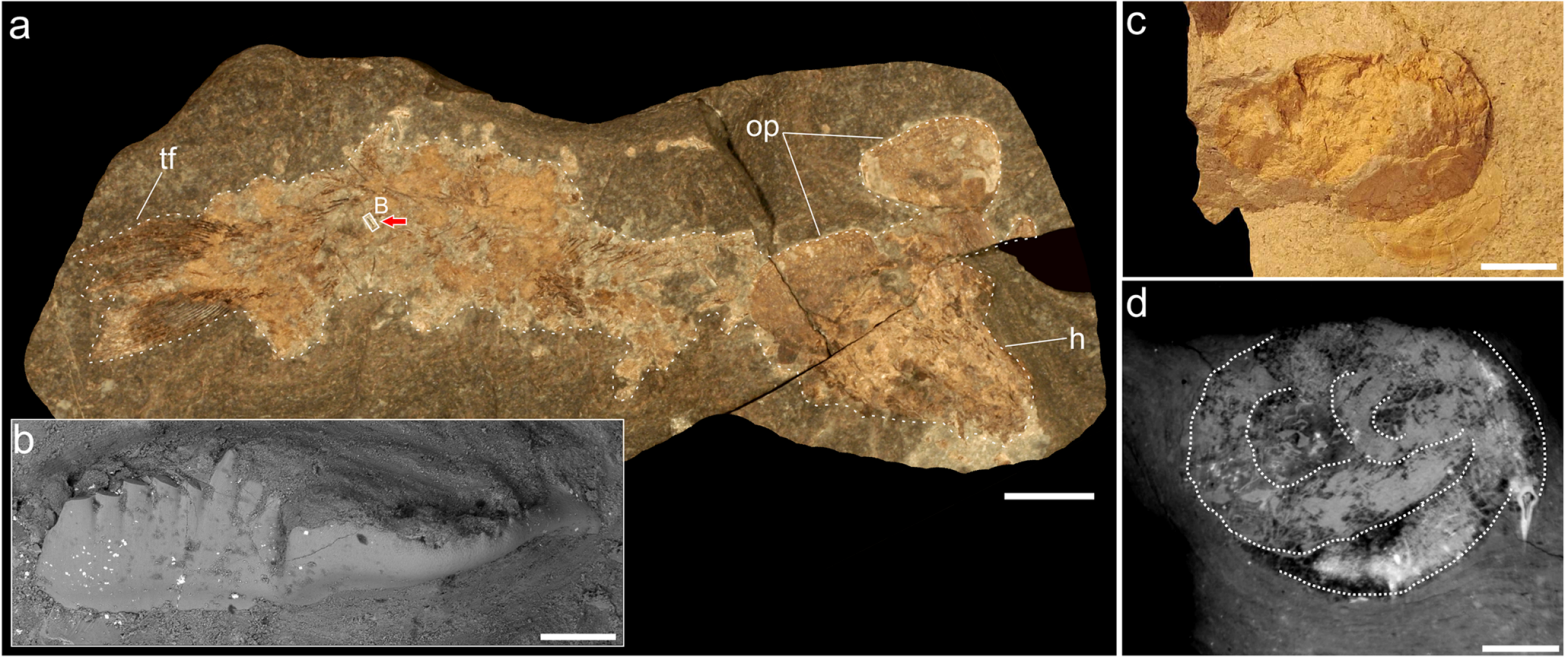
**a** Coelacanth fish, specimen GIUS 4–3654/1. **b** Conodont element found in its digestive tract. **c** Conodont-bearing, spiral coprolite specimen GIUS 4–3624/4. **d** Virtual thin section showing spiral internal organization of this specimen. *h* head, *op* opercula, *tf* tail fin. *Scale bars* 10 mm (**a**), 0.2 mm (**b**), and 3 mm (**c**–**d**)

In the present paper, we report the presence of a single conodont element from the gut content of a coelacanth specimen (Fig. 1a–b) from the Upper Devonian deposits of Poland. Additionally, several conodont elements have also been detected within a phosphatic and spiral coprolite found in the same deposits (Fig. 1c–d). The gut content, and possibly the coprolite, is the first direct evidence for feeding on conodont animals by Late Devonian coelacanth fishes.

## Material and methods

The coelacanth fish and conodont-bearing coprolite were found in the Upper Devonian (lower Famennian) marly shales outcropping in the Kowala quarry, Holy Cross Mountains, Poland. These deposits are already known to yield abundant phosphatized arthropod cuticles and coprolites (Zatoń et al. 2014; Zatoń and Rakociński 2014). Both the coelacanth fossil and the spiral coprolite were examined uncoated and under low vacuum condition using a Philips XL30 environmental scanning electron microscope (ESEM). Both the stomach content and the coprolite fossil inclusions (that were visible on the exterior) were documented using back-scattered electron (BSE) imaging. Additionally, in order to retrieve high-resolution, three-dimensional data from both the coprolite’s exterior and interior, it was scanned using propagation phase-contrast synchrotron microtomography (PPC-SRμCT) at beamline ID19 of the European Synchrotron Radiation Facility (ESRF), France (Supplementary info).

The specimens are housed at the Faculty of Earth Sciences, University of Silesia, Sosnowiec, Poland, abbreviated GIUS 4-3654/1 (coelacanth) and GIUS 4-3624/4 (coprolite).

## Results

The coelacanth found in the lower Famennian deposits is not well-preserved, but represents an articulated skeleton consisting of part and counterpart. The general characteristics of the skeleton, especially the morphology of the gular plates and opercula, suggest that the coelacanth may represent, or be closely related to, the genus *Diplocercides*, of which isolated remains are known from other Famennian localities in the Holy Cross Mountains (Szrek 2007). However, a more precise identification is difficult due to the poor preservation of the cranial skeleton. The length of the specimen is 15 cm from the tip of its snout to the posterior end of its caudal fin. The conodont element has been found embedded within a light-brown phosphatic mass occurring in the posterior part of the coelacanth (Fig. 1a). The conodont is represented by a platform element most likely representing the polygnathid genus *Ctenopolygnathus*. It is 1.5 mm long and its surface is well-preserved, lacking any signs of etching caused by digestive processes (Fig. 1b).

**Fig. 2:**
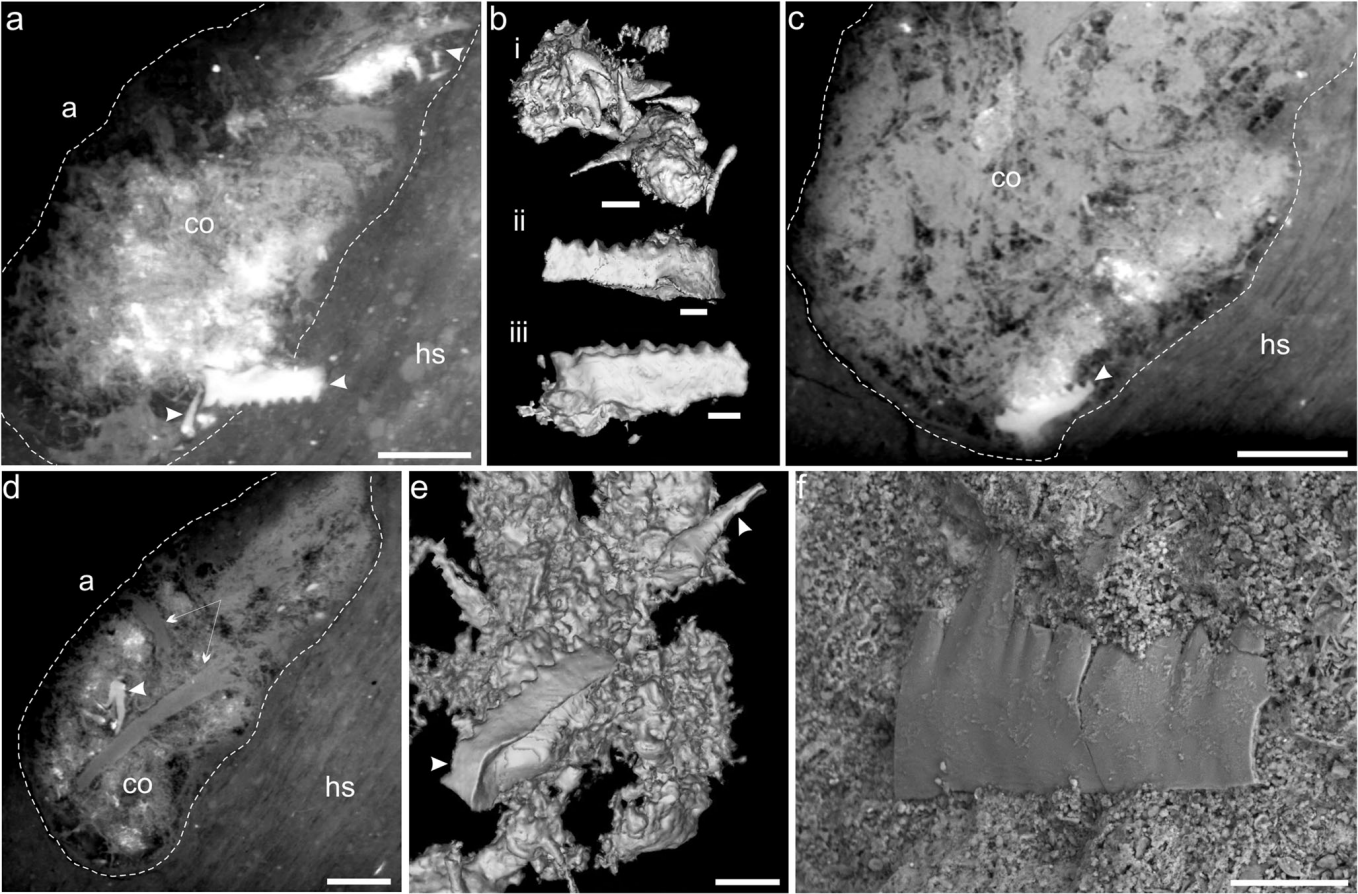
Conodont tooth elements found in the scanned coprolite. **a** Virtual thin section showing one pectiniform and several coniform conodont tooth elements (*white arrows*). **b** Segmented conodont tooth elements, where the lowest specimen is the same pectiniform element as shown in **a**. **c** Virtual thin section showing biomineralized structures and conodont tooth element (*white arrow*). **d** A virtual thin section of the coprolite showing dense accumulation of digested biomineralized structures, one pectiniform element (*white arrow*), and elongated enigmatic structures of possible conodont origin (*long arrows*). **e** Segmented conodont tooth elements and dense coprolite matrix, probably representing digested biomineralized tissues. **f** Polygnathid conodont element similar to *Mehlina kielcensis*. *co* coprolite mass, *hs* host sediment, *a* air. *Scale bars* 0.6 mm (**a**), 0.2 mm (*bi*), 0.15 mm (*bii*, *biii*), 0.5 mm (**c**), 0.85 mm (**d**), 0.25 mm (**e**), and 0.05 mm (**f**)

Synchrotron microtomography showed that the spiral coprolite (Fig. 1c–d) contains numerous conodont inclusions, including four pectiniform elements and many isolated coniform elements (Fig. 2b, e). The recognizable Pa-element is similar to the species *Mehlina kielcensis* (Fig. 2f). The conodont inclusions occur in a dense coprolite matrix (Fig. 2a, c–d) which in part probably represent other digested parts of the conodont jaw apparatus or digested biomineralized structures. Additionally, elongated, less dense structures with etched extremities occur in association with the conodont teeth (Fig. 2d). It is possible that these elements also originate from the jaw apparatus or other parts of the conodont animal, but poor preservation rules out more precise identification. Another coprolite (9.5 mm long and 8.3 mm wide) found in the same lower Famennian deposits at the Kowala quarry has previously been shown using ESEM to contain a single conodont element embedded within a phosphatic fecal mass (Zatoń and Rakociński 2014).

## Discussion

The presence of a conodont element within the Famennian coelacanth is not the result of any post-mortem process, as it is clearly embedded within the phosphatic mass occurring between the part and counterpart of the preserved fish. Therefore, its occurrence must reflect feeding on the conodont animal by the coelacanth. This is the first direct evidence of feeding on conodont animals by early coelacanth fishes, and also one of the few evidences of feeding on these animals in general. Previously, conodonts, belonging to two multielement taxa (*Oulodus* and *Icriodus*), have been found within the gut region of a palaeoniscoid fish from the Upper Devonian Gogo Formation in Australia by Nicoll (1977). In the same formation, Choo et al. (2009) found a basal actinopterygian fish *Gogosardina* with prioniodinid conodont elements preserved in its branchial regions. Williams (1990) noted conodont elements inside 9% of all specimens of *Cladoselache* sharks from the Famennian Cleveland Shale of Ohio. Conodont elements have also been found within the enigmatic metazoan called *Typhloesus wellsi* from the Carboniferous Bear Gulch Limestone in Montana (Conway Morris 1990).

Thus, the find not only supports the earlier notions that conodont animals, the extinct chordates (Purnell et al. 1995) which were particularly abundant in the Late Devonian seas (Dzik 2002), were ideal prey for various Devonian fishes, but also expands our knowledge about the diet of the Late Devonian coelacanths. However, the presence of only a single conodont element within the body cavity of a coelacanth does not provide any clue as to whether the fish actively hunted living conodont animals or scavenged carcasses lying on the seafloor. It is known that the living species *Latimeria chalumnae* is a predominantly piscivorous benthic or epibenthic feeder (Uyeno and Tsutsumi 1991). The stomach contents preserved in some fossil coelacanths, mainly consisting of arthropods (Lund and Lund 1984, 1985; Clement 2005), are also suggestive of feeding principally on benthic animals. Thus, we cannot exclude that the Late Devonian coelacanth from Kowala was a benthic scavenger. However, we must also bear in mind that early coelacanth species were morphologically very diverse (Friedman and Coates 2006; Wendruff and Wilson 2012) and thus their niche partitioning, along with feeding preferences, certainly varied as well.

Interestingly, the same lower Famennian, coelacanth-bearing deposits contain numerous fish coprolites. Recently, a coprolite containing a single conodont element has been found in the Kowala quarry by Zatoń and Rakociński (2014). Earlier, Dzik (2002) illustrated conodont elements aggregated together (probably representing coprolite contents) from the basal Famennian of Płucki, Holy Cross Mountains. Conodonts enclosed within phosphatic coprolites have also been noted in the lower Famennian Cleveland Shale in OH, USA by Williams (1990).

The new coprolite reported here contains several platform and conical conodont elements. Of course, identification of a producer based on the coprolite itself is always speculative. However, it is possible that at least some of the coprolites reported earlier by Zatoń and Rakociński (2014) and that presented here may have been produced by coelacanth fish, the disarticulated remains of which are common in the deposits investigated (Zatoń and Broda 2015). Apart from the supporting evidence in the form of a conodont element preserved within a coelacanth fish shown here, the spiral internal organization of the coprolite may be relevant (Fig. 1d). It is known that coelacanth fishes possess a spiral gut valve (Thoney and Hargis 1991). Moreover, the very small sizes of the lower Famennian coprolites from Kowala, which range only from 6 to 18 mm in length (Zatoń and Rakociński 2014), are comparable to the size of the coelacanth discussed.

The relative diversity of fishes from the lower Famennian (*crepida* conodont Zone) deposits may indicate coelacanths as producers of at least some of the coprolites. Other fish remains are known in these deposits. Sharks and acanthodians are known from their teeth and scales (Ginter 2002) and ganoid fishes as scales inside coprolites (Zatoń and Rakociński 2014). However, the coelacanth remains are the most numerous fossils occurring in these deposits. To date, incomplete coelacanth remains comprise 23 specimens represented either by isolated opercula or associated cranial elements (Zatoń and Broda 2015). Other coelacanth remains (*Diplocercides* sp.) have also been reported from deposits of the same conodont zone in the Wietrznia locality (Szrek 2007). Thus, it seems that the lower Famennian *crepida* conodont Zone deposits are particularly rich in coelacanths.

## Electronic supplementary material

ESM 1 (DOC 32 kb)
