# Supplementary material for: The first direct evidence of a Late Devonian coelacanth fish feeding on conodont animals
Source: Naturwissenschaften. 2017 Mar 10;104(3):26. doi: 10.1007/s00114-017-1455-7 (PMC5346137; doi:10.1007/s00114-017-1455-7)
Supplement: Supplementary file 1 — (DOC 32 kb) [file 114_2017_1455_MOESM1_ESM.doc]

Supplementary information

Methods

The coprolite was scanned in vertical series of 5 mm, in half acquisition mode (i.e. the center at rotation was set at the side of the camera field of view, resulting in a doubling of the reconstructed field of view). The propagation distance (i.e. the distance between the sample on the rotation stage and the camera) was set at 2800 mm. The camera was a sCMOS PCO edge 5.5 detector, which was mounted on an optical device resulting in an isotropic voxel size of 6.54 μm, and coupled to a 1000-μm thick GGG:Eu (Gadolinium gallium garnet doped with europium) scintillator. The beam was produced by a W150 wiggler (11 dipoles, 150mm period) with a gap of 48 mm and was filtered with 5.6 mm aluminum and 5 mm copper. The resulting detected spectrum had an average energy of 110.8 keV. Each sub scan was performed using 6000 projections of 0.03s each over 360 degrees. The reconstructions of the scanned data were based on a phase retrieval approach (Paganin et al. 2002; Sanchez et al. 2012; Qvarnström et al. in review) and ring artefacts were corrected using an in-house correction tool (Lyckegaard et al. 2011). Binned versions (bin2) were calculated to allow faster processing and screening of the samples since the full resolution data was large. The final volumes consist in stacks of 16 bits TIFF images that were subsequently imported and segmented in the software VGStudio MAX version 3.0 (Volume Graphics Inc.).

References

Lyckegaard A, Johnson G, Tafforeau P (2011) Correction of ring artifacts in X-raytomographic images. Int. J. Tomogr. Simul. 18:1–9.

Paganin D, Mayo SC, Gureyev TE, Miller PR, Wilkins SW (2002) Simultaneous phase and amplitude extraction from a single defocused image of a homogeneous object. J. Microsc. 206:33–40.

Sanchez S, Ahlberg PE, Trinajstic KM., Mirone A, Tafforeau P (2012) Three-dimensional synchrotron virtual paleohistology: a new insight into the world of fossil bone microstructures. Microsc. Microanal*.* 18:1095–1105.

Qvarnström M, Niedźwiedzki G, Tafforeau P, Žigaitė Ž, Ahlberg PE (in review) Synchrotron phase-contrast microtomography of coprolites generates novel palaeobiological data. Scientific Reports.
